# Editorial: Frailty and Sarcopenia in Various Cachectic Kidney Diseases, Volume II

**DOI:** 10.3389/fmed.2022.936512

**Published:** 2022-05-31

**Authors:** Yoshiyuki Morishita, Chia-Ter Chao

**Affiliations:** ^1^Division of Nephrology, Department of Integrated Medicine, Saitama Medical Center, Jichi Medical University, Saitama, Japan; ^2^Division of Nephrology, Department of Internal Medicine, National Taiwan University Hospital, Taipei, Taiwan; ^3^Division of Nephrology, Department of Internal Medicine, National Taiwan University College of Medicine, Taipei, Taiwan; ^4^Graduate Institute of Toxicology, National Taiwan University College of Medicine, Taipei, Taiwan

**Keywords:** kidney disease, frailty, sarcopenia, bone fracture, physical inactivity, mortality, CKD-related complication

Patients with chronic kidney disease (CKD) tend to develop degenerative syndromes, such as frailty, sarcopenia, polypharmacy, and cognitive impairment (1). The emergence of these syndromes predisposes these patients to a higher risk of mortality, functional decline, and an increased consumption of healthcare resources (2). The pathogenesis of frailty, sarcopenia, and other CKD-related complications is complex, ranging from an inherited susceptibility, epigenetic dysregulation, malnutrition-inflammation syndrome, multimorbidity, and most importantly, the adverse effects posed by uremic toxins (3). Regrettably, effective treatments against these degenerative syndromes in patients with CKD remain are unavailable, partly owing to the incomplete understanding of their underlying pathogenesis, and also the monotonous nature of the tested approaches. Therefore, following our first volume of a Research Topic on this topic, we curated a second volume to invite interested researchers for brainstorming about new directions in various aspects of frailty, sarcopenia, and other CKD-related complications. The compilation of selected articles in this Research Topic is expected to shed light on how we interpret and manage these syndromes in patients with CKD.

de Amorim et al. examined the prevalence and associated factors of sarcopenia in 139 non-dialysis patients with CKD in a prospective cross-sectional study. They found that the prevalence of sarcopenia was high in this population (sarcopenia: 20.9% [29/139], severe sarcopenia: 2.9% [4/13]). They also found that the phase angle, which was measured with bioelectrical impedance analysis, plasma interleukin-6 (IL-6) concentrations, and serum creatinine concentrations were independently associated with sarcopenia in this population. These results suggested that the phase angle, plasma IL-6 concentrations, and serum creatinine concentrations may be useful biomarkers to assess sarcopenia in this population.

Frailty is considered to increase the risk of bone fracture and mortality in dialysis-dependent patients with CKD (4, 5). Wu et al. investigated the usefulness of a fracture risk assessment tool and bone turnover markers, such as the serum procollagen type 1 amino-terminal propeptide concentration, serum C-terminal cross-linking telopeptide of type I collagen concentration, serum bone-specific alkaline phosphatase concentration, serum dickkopf-related protein concentration, and serum sclerostin concentration, as prediction markers for all-cause mortality and cardiovascular mortality in 164 dialysis-dependent patients with CKD in a prospective study. They found that a high risk of fracture detected by fracture risk assessment tool was independently associated with all-cause mortality but not cardiovascular morality. However, bone turnover markers concentrations were not associated with the mortality risk during a mean of 3.5 ± 1.0 years of follow-up. These results indicate that fracture risk assessment tool rather than bone turnover markers may be a useful tool for predicting the prognosis of this population.

Zhang, Ren, et al. conducted a systematic review and meta-analysis to investigate physical inactivity estimated by daily step counts in patients with CKD at a different stage. They found a trend in the number of daily steps in patients with CKD at different stages (post-transplantation > pre-dialysis > peritoneal dialysis > hemodialysis) by analyzing 28 previous studies. These results suggested that physical inactivity progresses in relation to the progression of severity of renal impairment. Another study aiming to investigate the association of physical inactivity and mortality in patients with CKD (including subgroup analysis of different CKD stage) is planned by Zhang, Wang, et al. This study may be useful for improving the prognosis of patients with CKD by improving physical inactivity.

In this Research Topic, several studies reported various pathological conditions that were frequently complicated by CKD. Chen et al. reported that the left ventricular end-diastolic and left ventricular end-systolic diameters estimated by an echocardiogram were predictors of changes in the left ventricular ejection fraction in 2,148 patients with heart failure and a left ventricular ejection fraction < 35%. Kaneko et al. reported that carnitine supplementation may improve the erythropoietin resistance index in 13 patients who underwent peritoneal dialysis. Matsuyama et al. reported that elobixibat improved constipation and lipid metabolism in 42 patients with CKD without serious adverse events. Elobixibat is a novel laxative that inhibits bile acid transporters of the terminal ileum, and increases the amount of bile acid flowing into the colon lumen and increases water secretion into the lumen of the large intestine. These studies should be confirmed by large-sized, randomized, controlled studies to establish new evidence.

Finally, Yanai et al. performed a translational study, which investigated the role of microRNA (miRNA) in age-dependent renal impairment. They found that serum miRNA-503-5p levels were decreased in patients with age-dependent renal impairment. However, the inhibition of miRNA-503-5p had no effects on age-dependent renal impairment, although it had therapeutic effects of renal fibrosis and glomerular sclerosis in animal models *in vivo*. These results indicate that miRNA-503-5p might be related to age-dependent renal impairment. An accumulation of further evidence is required to clarify molecules that are involved in age-dependent renal impairment.

In conclusion, interdisciplinary studies, involving clinical and basic studies, as in this Research Topic, are likely to substantially aid in the understanding of frailty and sarcopenia in various cachectic kidney diseases. However, further research is still required to extend our understandings in this area.

## Author Contributions

YM compiled the contributions from C-TC. All authors approved the final version of the manuscript.

## Conflict of Interest

The authors declare that the research was conducted in the absence of any commercial or financial relationships that could be construed as a potential conflict of interest.

## Publisher's Note

All claims expressed in this article are solely those of the authors and do not necessarily represent those of their affiliated organizations, or those of the publisher, the editors and the reviewers. Any product that may be evaluated in this article, or claim that may be made by its manufacturer, is not guaranteed or endorsed by the publisher.
